# Shorter serial intervals in SARS-CoV-2 cases with Omicron BA.1 variant compared with Delta variant, the Netherlands, 13 to 26 December 2021

**DOI:** 10.2807/1560-7917.ES.2022.27.6.2200042

**Published:** 2022-02-10

**Authors:** Jantien A Backer, Dirk Eggink, Stijn P Andeweg, Irene K Veldhuijzen, Noortje van Maarseveen, Klaas Vermaas, Boris Vlaemynck, Raf Schepers, Susan van den Hof, Chantal BEM Reusken, Jacco Wallinga

**Affiliations:** 1 Center for Infectious Disease Control, WHO COVID-19 reference laboratory, National Institute for Public Health and the Environment (RIVM), Bilthoven, The Netherlands; 2 Saltro Diagnostic Center for Primary Care, Utrecht, The Netherlands; 3 Department of Medical Microbiology, University Medical Center Utrecht, Utrecht, the Netherlands; 4 SYNLAB, Heppignies, Belgium; 5 Department of Biomedical Data Sciences, Leiden University Medical Center, Leiden, The Netherlands

**Keywords:** SARS-CoV-2, COVID-19, Omicron variant, serial interval, Incubation period, transmission pairs

## Abstract

The SARS-CoV-2 Omicron variant has a growth advantage over the Delta variant because of higher transmissibility, immune evasion or shorter serial interval. Using S gene target failure (SGTF) as indication for Omicron BA.1, we identified 908 SGTF and 1,621 non-SGTF serial intervals in the same period. Within households, the mean serial interval for SGTF cases was 0.2–0.6 days shorter than for non-SGTF cases. This suggests that the growth advantage of Omicron is partly due to a shorter serial interval.

The Omicron variant (Phylogenetic Assignment of Named Global Outbreak (Pango) lineage designation: B.1.1.529) of severe acute respiratory syndrome coronavirus 2 (SARS-CoV-2) was first reported by South Africa on 24 November 2021 and designated by the World Health Organization as a variant of concern on 26 November 2021 [1]. It is characterised by a fast epidemic growth relative to the Delta (B.1.617.2) variant [2]. Several epidemiological factors may contribute to the fast relative growth rate of this new variant: (i) immune evasion [3-5], (ii) higher intrinsic transmission potential [6] (an increase in the basic reproduction number, defined as the average number of secondary cases generated by an infectious individual in a susceptible population) and (iii) a shorter serial interval (i.e. the duration of time between symptom onset of a case and its infector). A variant with a shorter serial interval than another variant with the same reproduction number would have an increased epidemic growth rate. While early reports provide evidence for substantial immune evasion and suggest an increased transmission potential [3-6], little is known about the serial interval of the Omicron variant. We assess whether the serial intervals of the Omicron BA.1 and Delta variant differ by comparing transmission pairs of both variants during the same time period.

## Identification of serial intervals by variant

The Omicron BA.1 variant was first identified in the Netherlands in a case whose sample was obtained on 19 November 2021. Symptom onset dates and postal codes of diagnosed SARS-CoV-2 cases are reported to a national surveillance database. If an infector of the case has been identified through source and contact tracing, a unique identifier for this infector is reported as well. We identified pairs of primary and secondary cases from this national surveillance database and measured the serial interval as difference between symptom onset day of a case and their infector.

A fraction of the cases reported in the national surveillance database were tested in two laboratories that analyse specimens with the TaqPath COVID-19 RT-PCR Kit (ThermoFisher Scientific, Waltham MA, United States). This PCR kit targets three genes. Failure of the probe targeting the S gene, while the ORF1ab and N probe result in a proper signal (S gene target failure (SGTF), also referred to as S dropout) identifies the presence of a deletion in the S gene (spike amino acid residues Δ69–70) which has been associated with the Omicron BA.1 but not the Delta variant. Non-SGTF is highly predictive of the Delta variant and SGTF is highly predictive for the Omicron BA.1 variant during times with little to no circulation of other variants [4]. In samples with lower viral loads, SGTF allocation is less accurate as the S gene target is the least sensitive target of the three genes. Therefore, a stringent threshold of ≤ 30 quantification cycle (Cq) values was used on the ORF1ab and N targets for inclusion in further analyses.

We included transmission pairs with a minimum serial interval of −5 days and a maximum serial interval of 15 days. Among those, we included transmission pairs with a symptom onset date for the infector between 13 and 26 December 2021 (week 50 and 51), as reported by 24 January 2022, and report our results by week. The overall share of Omicron variant BA.1 detected in test-positive cases in the Netherlands was 9.0% in week 50 and 28.6% in week 51 [7]. We followed a cohort approach to minimise the impact of data truncation and differences in epidemic growth by variant on the outcome. Most cases in the national surveillance database were tested and reported within 5 days after symptom onset. Combined with a maximum serial interval of 15 days, this would mean that a secondary case of the cohort would be tested and reported at the time the data were retrieved from the notification system. To ensure independent serial intervals, we included only unique infectors by choosing one of their cases at random. We excluded transmission pairs where infector or case had a missing postal code, where both infector and case lacked SGTF results, or where infector and case had differing SGTF results.

We will refer to transmission pairs with an SGTF case or an SGTF infector as SGTF transmission pairs, and to transmission pairs with a non-SGTF case or a non-SGTF infector as non-SGTF transmission pairs. We will refer to transmission pairs with a case and infector with the same postal code as within-household transmission pairs, and to transmissions with a case and infector with a different postal code as between-household transmission pairs, because 97% of transmission pairs with identical postal code live within the same household [8].

## Observed serial intervals

In week 50 (13–19 December 2021) we identified 235 SGTF transmission pairs and 919 non-SGTF transmission pairs, excluding 14 pairs with opposing SGTF results, eight pairs without postal code, 193 pairs with non-unique infectors and six pairs with a serial interval outside the range of −5 to 15 days. The mean serial interval of 3.5 days (standard deviation (SD): 2.4 days) for the 164 SGTF within-household pairs was significantly shorter than the mean serial interval of 4.1 days (SD: 2.8 days) for the 761 non-SGTF within-household pairs (Figure, bootstrapped p value = 0.0026). A similar but not significant difference was found between the mean serial interval of 3.3 days (SD: 2.4 days) for the 71 SGTF between-household pairs and the mean serial interval of 3.5 (SD: 2.8 days) days for the 158 non-SGTF between-household pairs (bootstrapped p value = 0.24). We grouped the within-household transmission pairs by the vaccination status of the infector and the case and found that for each group, the mean serial interval for SGTF transmission pairs was smaller than for non-SGTF transmission pairs, with statistical significance of this difference only when the infector was fully vaccinated (see Supplementary Figure S1 for empirical cumulative density functions by vaccination status of infector and infectee).

**Figure:**
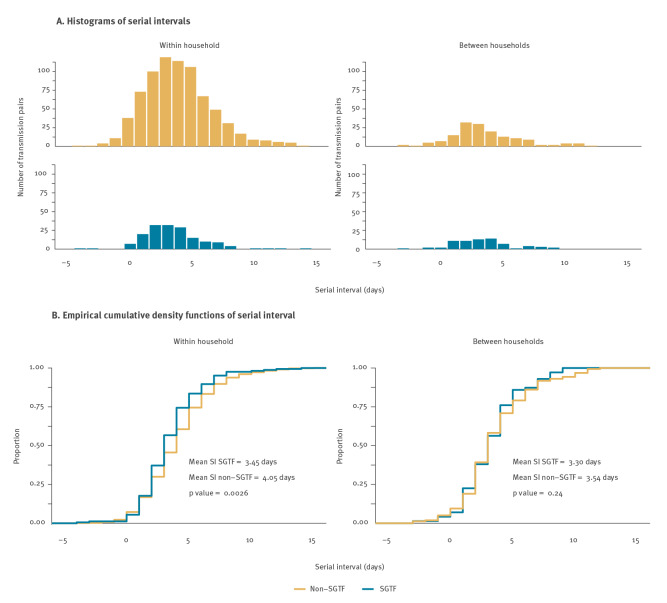
Observed distribution of serial interval of SARS-CoV-2 transmission pairs with infectors having their symptom onset date in week 50 (13–19 December 2021), the Netherlands (n = 1,154)

SARS-CoV-2: severe acute respiratory syndrome coronavirus 2; SGTF: S gene target failure; SI: serial interval.

The data for this analysis are provided in Supplementary Table S1. Significance of having different mean serial intervals is tested by bootstrapping.

In week 51 (20–26 December 2021), we identified 673 SGTF transmission pairs and 702 non-SGTF transmission pairs, excluding 13 pairs with opposing SGTF results, nine pairs without postal code, 239 pairs with non-unique infectors, and two pairs with a serial interval outside the range of −5 to 15 days. Also in that week, the mean serial interval of 3.0 days (SD: 2.3 days) for the 480 SGTF within-household pairs was shorter than the mean serial interval of 3.2 days (SD: 2.6 days) for the 572 non-SGTF within-household pairs (bootstrapped p value = 0.084). A slightly shorter serial interval for SGTF vs non-SGTF was observed for between-household pairs (a detailed presentation of the observed serial intervals in week 51 is provided in Supplementary Figure S2).

## Incubation period

In addition to the transmission pairs, we studied cases with known exposure information that allowed us to infer the incubation period [9]. We identified 258 SGTF cases and 255 non-SGTF cases with reported symptom onset date between 1 December 2021 and 2 January 2022 (i.e. 13% of all cases with known exposure information in that period). The mean incubation period is estimated to be 3.2 days (SD: 2.2 days) for SGTF cases and 4.4 days (SD: 2.5 days) for non-SGTF cases, with non-overlapping credible intervals (Supplementary Figure S3 and Table S2).

## Discussion

This early investigation offers evidence to support a shorter serial interval among the SGTF transmission pairs presumed to be caused by the Omicron variant as compared with the non-SGTF transmission pairs presumed to be caused by the Delta variant. This lends support to the hypothesis that the recent rapid growth of the Omicron BA.1 variant was in part driven by a shortened serial interval as compared with infections with the Delta variant. The observed difference of 0.2–0.6 days is in line with the difference in the incubation period between the two variants.

During the study period, the contact tracing guidelines differed for the two variants regarding contacts outside the household. Until 23 December 2021, guidelines for the Omicron variant were stricter than for Delta, with longer isolation and quarantine periods and requiring quarantine also for fully vaccinated or recently recovered contacts. These differences may offer a possible explanation for the observed shorter serial interval of the SGTF transmission pairs between households compared with within households. However, these differences do not explain the observed shorter serial interval of the SGTF transmission pairs within households and the shorter incubation period of SGTF cases. Therefore, the observed difference in between-household pairs is also expected to be due to the difference in variants.

To generalise the observed differences between serial interval for SGTF and non-SGTF transmission pairs, proper control for the control measures in place and other confounding factors such as age and vaccination status of the cases and their infectors are required. The difference in the duration of serial interval between successive weeks suggest a possible effect of changing control measures (a complete lockdown was implemented on 19 December 2021) and of subsequently changing behaviour, including increased testing around the Christmas period.

The reported values of the mean serial interval of 3.5 and 3.0 days for the Omicron variant are a bit longer than tentative estimates reported previously. Mean serial intervals of 2.2 days and 2.8 days (range: 1–7 days) were reported for an outbreak in South Korea [10,11]. Kremer et al. report a mean serial interval of 2.75 days for the Omicron variant and 3.00 days for the Delta variant in Belgium [12]. These values are more in line with the mean serial interval of 3.0 days that we observed for the period of 20–26 December 2021 in the Netherlands. The estimated value of the median incubation period of 2.8 days for the Omicron variant is in line with earlier estimates. A median incubation period of 3 days for Omicron was reported for a superspreading event in Norway [13] and for a cluster in Nebraska [14]. Although not all of these earlier reports allowed for a direct comparison between the reported values for the mean serial interval and the median incubation period between the Omicron and Delta variant in the same period, the low values suggest that also in these settings, the serial interval and the incubation time of the Omicron BA.1 variant are shorter than those for the Delta variant. A short serial interval and a short incubation period will make timely contact tracing more challenging, which will have a negative impact on reducing onward transmission [15].

There are indications for a potential different place of replication in the host and a different route of entry for the Omicron variant, which suggests a mechanism to account for a shorter serial interval and a shorter incubation period [6,16]. Further studies that include the viral load and shedding dynamics relative to the symptom onset date of the primary case are crucial.

## Conclusion

A short serial interval offers, next to immune evasion and higher transmissibility, an explanation for the growth advantage of the Omicron BA.1 variant over the Delta variant. This leads to a faster succession of infection generations. Mitigating the observed rapid spread of the SARS-CoV-2 Omicron variant will therefore continue to require multilayered interventions such as case finding and contact tracing, as well as booster vaccination and non-pharmaceutical interventions.

## Supplementary Data

543000 Supplement

## References

[1] World Health Organization (WHO). Tracking SARS-CoV-2 variants. Geneva: WHO. [Accessed: 8 Feb 2022]. Available from: https://www.who.int/en/activities/tracking-SARS-CoV-2-variants

[2] Ito K Piantham C Nishiura H . Relative instantaneous reproduction number of Omicron SARS-CoV-2 variant with respect to the Delta variant in Denmark. J Med Virol. 2021. 34967453 10.1002/jmv.27560 PMC9015237

[3] Cele S Jackson L Khan K Khoury DS Moyo-Gwete T Tegally H SARS-CoV-2 Omicron has extensive but incomplete escape of Pfizer BNT162b2 elicited neutralization and requires ACE2 for infection. medRxiv. 2021 .12.08.21267417 10.1101/2021.12.08.21267417

[4] Eggink D Andeweg SP Vennema H van Maarseveen N Vermaas K Vlaemynck B Increased risk of infection with SARS-CoV-2 Omicron BA.1 compared with Delta in vaccinated and previously infected individuals, the Netherlands, 22 November 2021 to 19 January 2022. Euro Surveill. 2022;27(4):2101196. 10.2807/1560-7917.ES.2022.27.4.2101196 35086609 PMC8796294

[5] Wilhelm A Widera M Grikscheit K Toptan T Schenk B Pallas C Reduced neutralization of SARS-CoV-2 Omicron variant by vaccine sera and monoclonal antibodies. medRxiv. 2021 .12.07.21267432 10.1101/2021.12.07.21267432

[6] Peacock TP Brown JC Zhou J Thakur N Newman J Kugathasan R The SARS-CoV-2 variant, Omicron, shows rapid replication in human primary nasal epithelial cultures and efficiently uses the endosomal route of entry. bioRxiv. 2021 .12.31.474653 . 10.1101/2021.12.31.474653

[7] Rijksinstituut voor Volksgezondheid en Milieu (RIVM). Varianten van het coronavirus SARS-CoV-2. [Variants of the coronavirus SARS-CoV-2]. Bilthoven: RIVM. [Accessed: 27 Jan 2022]. Dutch. Available from: https://www.rivm.nl/coronavirus-covid-19/virus/varianten

[8] Centraal Bureau voor de Statistiek (CBS) Transmissieparen adres, werkgever en school, jan-sep 2021. [Transmission pairs' address, employer and school, Jan-Sep 2021]. Den Haag: CBS, 2021. Available from: https://www.cbs.nl/nl-nl/maatwerk/2021/49/transmissieparen-adres-werkgever-en-school-jan-sep-2021

[9] Backer JA Klinkenberg D Wallinga J . Incubation period of 2019 novel coronavirus (2019-nCoV) infections among travellers from Wuhan, China, 20-28 January 2020. Euro Surveill. 2020;25(5). 10.2807/1560-7917.ES.2020.25.5.2000062 32046819 PMC7014672

[10] Kim D Jo J Lim J-S Ryu S . Serial interval and basic reproduction number of SARS-CoV-2 Omicron variant in South Korea. medRxiv. 2021 .12.25.21268301 . 10.1101/2021.12.25.21268301 PMC8948735 35336939

[11] Lee JJ Choe YJ Jeong H Kim M Kim S Yoo H Importation and transmission of SARS-CoV-2 B.1.1.529 (Omicron) variant of concern in Korea, November 2021. J Korean Med Sci. 2021;36(50):e346. 10.3346/jkms.2021.36.e346 34962117 PMC8728587

[12] Kremer C Braeye T Proesmans K André E Torneri A Hens N . Observed serial intervals of SARS-CoV-2 for the Omicron and Delta variants in Belgium based on contact tracing data, 19 November to 31 December 2021. medRxiv. 2022.01.28.22269756 . 10.1101/2022.01.28.22269756 PMC9328897 35732195

[13] Brandal LT MacDonald E Veneti L Ravlo T Lange H Naseer U Outbreak caused by the SARS-CoV-2 Omicron variant in Norway, November to December 2021. Euro Surveill. 2021;26(50). 10.2807/1560-7917.ES.2021.26.50.2101147 34915975 PMC8728491

[14] Jansen L Tegomoh B Lange K Showalter K Figliomeni J Abdalhamid B Investigation of a SARS-CoV-2 B.1.1.529 (Omicron) Variant Cluster - Nebraska, November-December 2021. MMWR Morb Mortal Wkly Rep. 2021;70(5152):1782-4. 10.15585/mmwr.mm705152e3 34968376 PMC8736273

[15] Kucharski AJ Klepac P Conlan AJK Kissler SM Tang ML Fry H Effectiveness of isolation, testing, contact tracing, and physical distancing on reducing transmission of SARS-CoV-2 in different settings: a mathematical modelling study. Lancet Infect Dis. 2020;20(10):1151-60. 10.1016/S1473-3099(20)30457-6 32559451 PMC7511527

[16] Kozlov M . Omicron’s feeble attack on the lungs could make it less dangerous. Nature. 2022;601(7892):177. 10.1038/d41586-022-00007-8 34987210

