# Supplementary material for: Shorter serial intervals in SARS-CoV-2 cases with Omicron BA.1 variant compared with Delta variant, the Netherlands, 13 to 26 December 2021
Source: Euro Surveill. 2022 Feb 10;27(6):2200042. doi: 10.2807/1560-7917.ES.2022.27.6.2200042 (PMC8832521; doi:10.2807/1560-7917.ES.2022.27.6.2200042)
Supplement: Supplement [file 22-00042_BACKER_Supplement.pdf]

This supplementary material is hosted by Eurosurveillance as supporting information alongside the article “Shorter serial intervals in SARS-CoV-2 cases with Omicron BA.1 variant compared with Delta variant, the Netherlands, 13 to 26 December 2021”, on behalf of the authors, who remain responsible for the accuracy and appropriateness of the content. The same standards for ethics, copyright, attributions and permissions as for the article apply. Supplements are not edited by Eurosurveillance and the journal is not responsible for the maintenance of any links or email addresses provided therein.

Supplementary material to

Shorter serial intervals in SARS-CoV-2 cases with Omicron BA.1 variant compared with Delta variant, the Netherlands, 13 to 26 December 2021

Jantien A. Backer[1], Dirk Eggink[1], Stijn P. Andeweg[1], Irene K. Veldhuijzen[1], Noortje van Maarseveen[2,3], Klaas Vermaas[2], Boris Vlaemynck[4], Raf Schepers[4], Susan van den Hof[1], Chantal B.E.M. Reusken[1], Jacco Wallinga[1,5]

[1] Center for Infectious Disease Control, WHO COVID-19 reference laboratory, National Institute for Public Health and the Environment (RIVM), Bilthoven, The Netherlands

[2] Saltro Diagnostic Center for Primary Care, Utrecht, The Netherlands.

[3] Department of Medical Microbiology, University Medical Center Utrecht, Utrecht, the Netherlands

[4] SYNLAB, Heppignies, Belgium

[5] Department of Biomedical Data Sciences, Leiden University Medical Center, Leiden, The Netherlands

Data

Table S1 Number of observed transmission pairs by serial interval, study week, type (within or between households) and SGTF result; data shown in Figure 1 in main text for week 50 and in Figure S2 for week 51.

	Week 50 (13-19 December 2021)				Week 51 (20-26 December 2021)			
	Within-household		Between households		Within-household		Between households	
**Serial interval (days)**	**non-SGTF**	**SGTF**	**non-SGTF**	**SGTF**	**non-SGTF**	**SGTF**	**non-SGTF**	**SGTF**
-5	0	0	0	0	3	0	0	0
-4	1	1	0	0	2	0	0	0
-3	1	1	2	1	3	4	1	0
-2	4	0	1	0	11	2	2	1
-1	11	0	5	2	8	8	3	4
0	38	7	7	2	38	31	8	12
1	73	20	15	11	64	64	17	35
2	100	32	32	11	99	110	23	39
3	119	32	30	13	109	95	31	37
4	114	29	20	14	78	65	17	25
5	106	15	13	7	60	42	10	20
6	67	10	11	1	41	27	13	11
7	49	9	9	4	24	11	2	6
8	31	4	2	3	17	9	2	2
9	17	0	2	2	7	7	0	0
10	9	1	4	0	2	1	1	0
11	8	1	4	0	3	0	0	0
12	6	1	1	0	0	2	0	0
13	5	0	0	0	1	1	0	1
14	1	1	0	0	1	1	0	0
15	1	0	0	0	1	0	0	0
**total**	761	164	158	71	572	480	130	193

Serial interval of within-household transmission pairs in week 50 (13 - 19 December 2021), by vaccination status

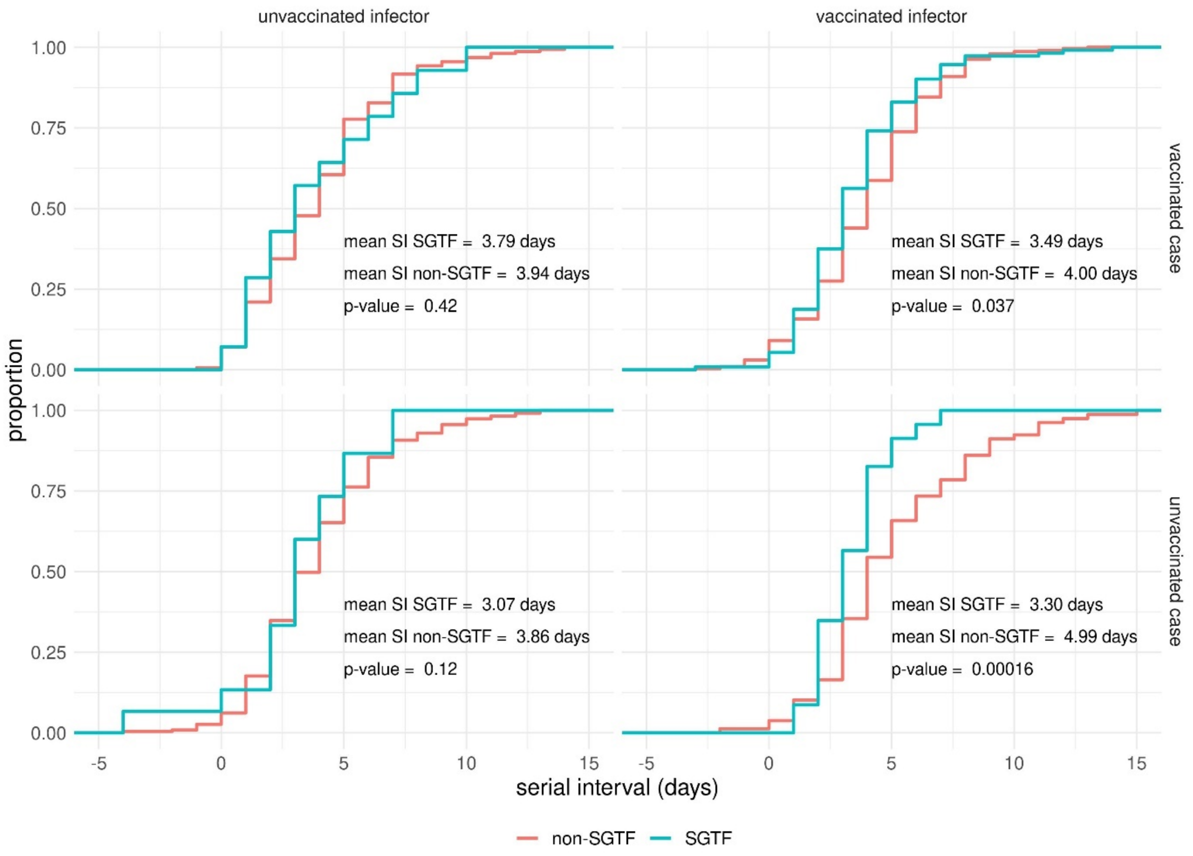

Figure S1 Observed distribution of serial interval of SARS-CoV-2 within-household transmission pairs with infectors having their symptom onset date in week 50 (13 - 19 December 2021) in The Netherlands, by vaccination status of the infector and case. A vaccinated status encompasses fully vaccinated persons including those who received a booster vaccination; an unvaccinated status encompasses unvaccinated and partially vaccinated persons. The panels show the difference between the empirical cumulative density functions for SGTF and non-SGTF transmission pairs by vaccination status of the index (columns) and case (rows). Significance of having different mean serial intervals is tested by bootstrapping.

Serial interval for week 51 (20 – 26 December 2021)

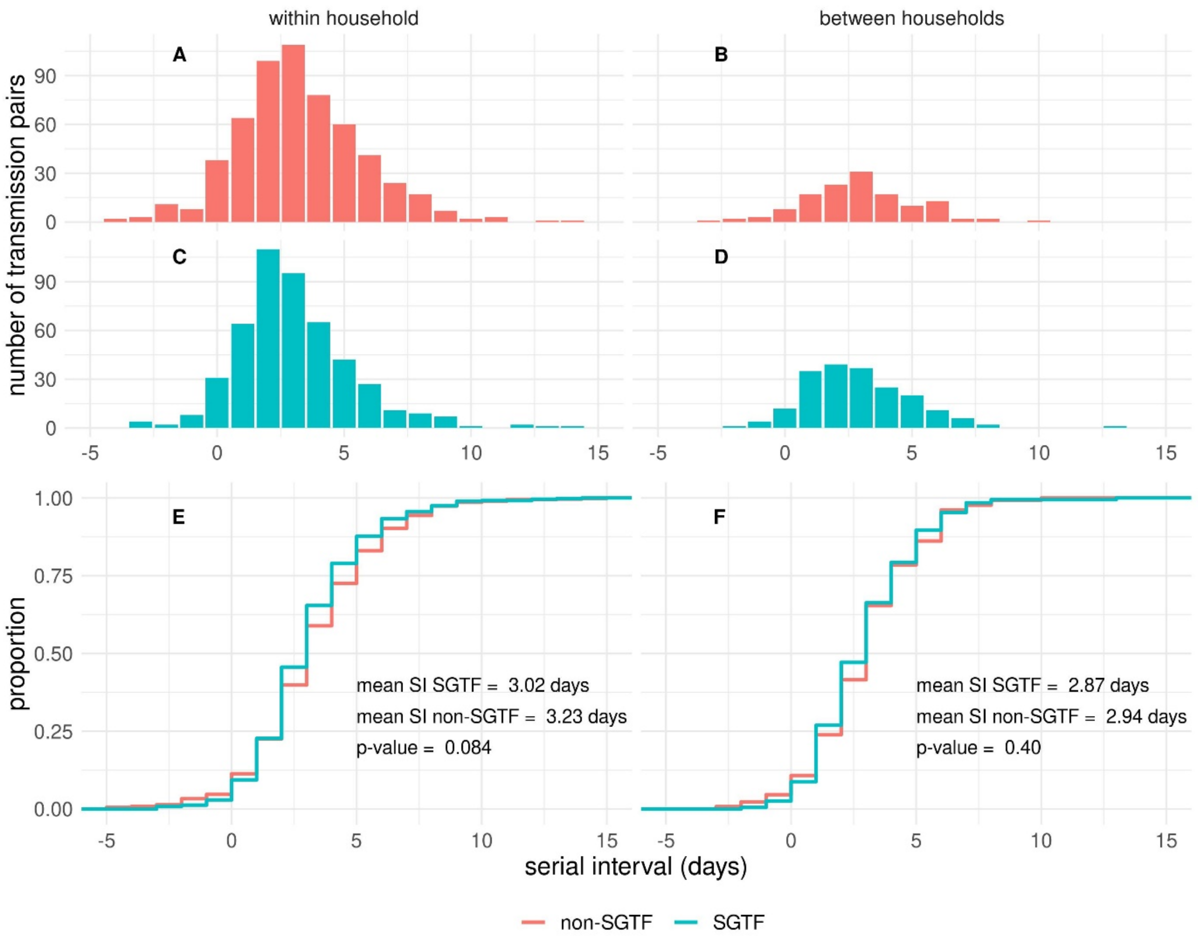

Figure S2 Observed distribution of serial interval of SARS-CoV-2 transmission pairs with infectors having their symptom onset date in week 51 (20 - 26 December 2021) in The Netherlands, (A) for non-SGTF within-household transmission pairs, (B) non-SGTF between-household transmission pairs, (C) SGTF within-household transmission pairs, (D) SGTF between-household transmission pairs, and the difference between the empirical cumulative density functions for SGTF and non-SGTF transmission pairs (E) within households and (F) between households (see Appendix for data). Significance of having different mean serial intervals is tested by bootstrapping.

Incubation period

For 258 SGTF cases and 255 non-SGTF cases, with a symptom onset date between 1 December 2021 and 2 January 2022, the exposure window and symptom onset are reported. The parameters are estimated using a previously published method (Backer et al., Euro Surveill., 2020). A Weibull distribution was found to best fit the data compared to gamma and lognormal distributions. The incubation period for SGTF cases are shorter than for non-SGTF cases and the credible intervals are non-overlapping (Fig. S3 and Tab. S2).

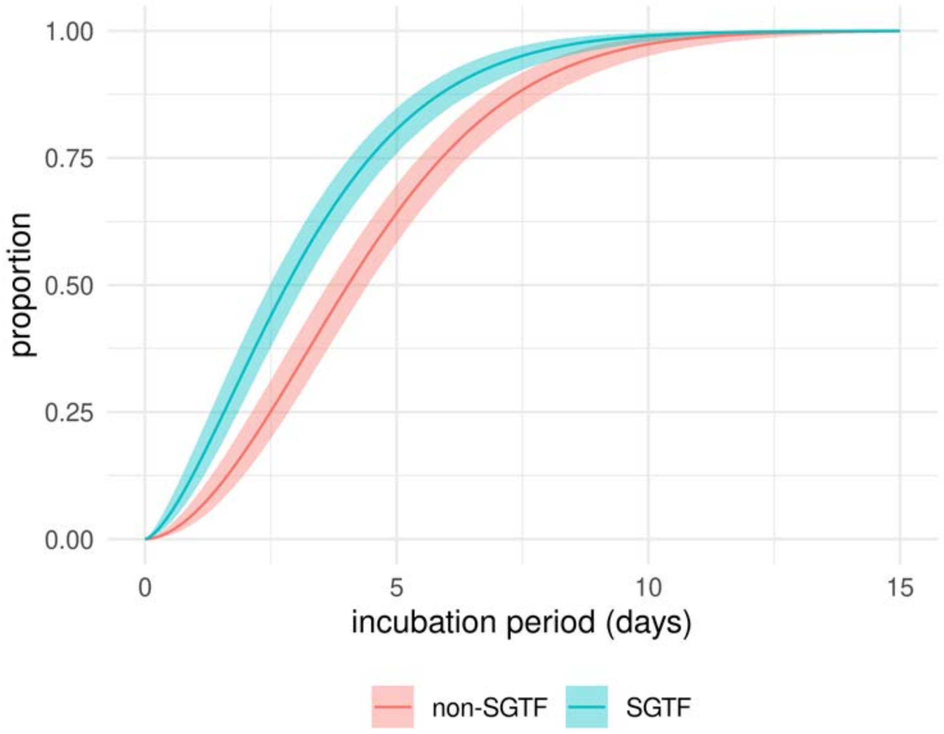

Figure S3 Estimated cumulative density function of the incubation period for 258 SGTF cases and 255 non-SGTF cases, with a symptom onset date between 1 December 2021 and 2 January 2022.

Table S2 Posterior parameter estimates for Weibull distributed incubation period

	non-SGTF		SGTF	
	median	95% cred. int.	median	95% cred. int.
Scale parameter	4.93	4.51 – 5.37	3.60	3.23 – 3.98
Shape parameter	1.83	1.59 – 2.08	1.50	1.32 – 1.70
Median (days)	4.0	3.6 – 4.4	2.8	2.5 – 3.2
Mean (days)	4.4	4.0 – 4.8	3.2	2.9 – 3.6
SD (days)	2.5	2.2 – 2.9	2.2	1.9 – 2.5
